# Treatment of post-prostatectomy urinary incontinence and erectile dysfunction: there is insufficient utilisation of care in German cancer survivors

**DOI:** 10.1007/s00345-020-03526-z

**Published:** 2020-12-01

**Authors:** Martin Baunacke, Maria-Luisa Schmidt, Christer Groeben, Angelika Borkowetz, Christian Thomas, Rainer Koch, Falk Hoffmann, Felix K. H. Chun, Lothar Weissbach, Johannes Huber

**Affiliations:** 1 Department of Urology, TU Dresden, Fetscherstrasse 74, 01307 Dresden, Germany; 2 Department of Health Services Research, Carl Von Ossietzky University, Ammerlaender Heerstrasse 140, 26111 Oldenburg, Germany; 3 Department of Urology, Goethe-University Hospital, Theodor-Stern-Kai 7, 60590 Frankfurt, Germany; 4 Health Research for Men gGmbH, Gfm, Claire-Waldoff-Strasse 3, 10117 Berlin, Germany

**Keywords:** Prostatectomy, Erectile dysfunction, Incontinence, Health services research

## Abstract

**Purpose:**

Treatment of post-prostatectomy urinary incontinence (UI) and erectile dysfunction (ED) increases quality of life (QoL). Aim of our study was to evaluate the utilisation of care among patients with post-prostatectomy UI and ED in Germany.

**Methods:**

The HAROW study documented treatment of patients with localised prostate cancer (≤ T2c) in Germany. 1260 patients underwent radical prostatectomy (RP). Patients answered validated questionnaires after a median follow-up of 6.3 years. Response rate was 76.8%.

**Results:**

Median age at RP was 65 (IQR 60–69) years. 14% (134/936) used more than one pad per day for UI. 25% (26/104, 30 missing) of UI patients underwent surgery to improve continence. Of patients without surgery, 41% (31/75) reported a moderate-to-severe issue concerning their incontinence with worse mental health and QoL. 81% (755/936) patients were unable to have an erection firm enough for sexual intercourse. Of all ED patients, 40% (319/793) used ED treatment regularly or tried it at least once. 49% (243/499) of patients with interest in sex never tried ED treatment. In multivariate analysis, patients not using ED treatments were older (≥ 70 years OR 4.1), and more often had preoperative ED (OR 2.3) and less interest in sex (OR 2.2). Nevertheless, 30% (73/240) of these patients had moderate-to-severe issues with their ED reporting worse mental health and QoL.

**Conclusion:**

Almost half of the patients without post-prostatectomy UI and ED treatment reported moderate-to-severe issues with a significant decrease in QoL. This indicates an insufficient utilisation of care in Germany.

**Electronic supplementary material:**

The online version of this article (10.1007/s00345-020-03526-z) contains supplementary material, which is available to authorized users.

## Introduction

Urinary incontinence (UI) and erectile dysfunction (ED) are the two most relevant side effects after radical prostatectomy (RP). Reported UI rates after RP vary between four and 40% [1], and ED rates vary between 10 and 69% [2]. Both functional outcomes are of critical importance for future quality of life [3].

In the majority of patients, UI can be treated by conservative and surgical treatment. For patients with persistent incontinence after one year of conservative treatment, including treatment with drugs, a surgical intervention can be used to improve their urinary continence [4]. There is a wide range of surgically implantable devices, including adjustable and non-adjustable male slings as well as artificial sphincters [5]. The artificial sphincter, being the most invasive surgical procedure, is the gold standard for the treatment of UI after RP [6].

Oral phosphodiesterase type 5 (PDE5) inhibitors represent the standard pharmacologic treatment for ED. Escalating ED treatment comprises intraurethral medication, penile injection therapy, vacuum erection devices, and penile prostheses [7]. Penile prostheses are the only surgical treatment option and show high patient satisfaction in a selected population [8].

Because poor functional outcomes after RP are burdensome, it is important to offer patients adequate and effective treatment. However, despite the great variety of therapy options for UI and ED, to date, little is known about the utilisation of available treatments in most healthcare systems. The aim of our study was to evaluate the utilisation of available treatments for post-prostatectomy incontinence and ED in a large cohort of patients receiving routine care in Germany.

## Materials and methods

The HAROW study (Hormone Therapy, Active Surveillance, Radiation, Operation, or Watchful Waiting) was a prospective observational noninterventional health services research study in Germany from 2008–2013 that evaluated the treatment of patients with histologically confirmed localised prostate cancer (T1a-T2c/N0/M0) [9]. Of this cohort, 1260 patients underwent radical prostatectomy. To evaluate the utilisation of treatment for post-prostatectomy UI and ED, we sent questionnaires to the participants by mail in Feb 2017. Non-responders were contacted by phone.

To evaluate the utilisation of available treatments for post-prostatectomy incontinence and ED, we analysed total utilisation of these treatments. We further analysed patients not using treatments regarding their level of burden because of their impairment and influence on quality of life. For ED, we analysed the subgroup of patients who were still interested in sex.

For these evaluations, we asked about use of ED treatment and interventions to improve continence. For ED treatment, we asked for use of PDE5 inhibitors, intraurethral medications, penile injection therapy, vacuum erection devices and penile prosthesis. Interventions to improve continence included slings, systems (ATOMS or ProAct), artificial sphincters, and other surgical procedures. Moreover, we evaluated d’Amico risk classification and Charlson score. Different validated questionnaires were used to measure functional outcome: The EPIC-26 questionnaire identified the extent of incontinence and urinary symptoms [10]. We defined UI by the use of two or more pads per day or having undergone incontinence surgery. Sexual function was validated according to ICHOM standards [11] with two additional questions from the EORTC QLQ-PR25 questionnaire [12] and one validated item on the use of erectile dysfunction aids [13]. Potency was defined by an erection firm enough for sexual intercourse without reporting ED treatment. Interest in sex was defined by small to large interest in sex according the EORTC QLQ-PR25 questionnaire [12]. Four items from the EORTC-QOL-30 questionnaire [14] were used to evaluate the overall quality of life and social sequelae. We used the PHQ-4 to evaluate mental health [15]. Furthermore, we assessed the patient’s Internet use concerning health issues. We applied the Chi-square test, the Mann–Whitney *U* test, and multivariate logistic analyses. *p* < 0.05 was considered to indicate significance. All calculations were performed with “IBM SPSS Statistics 22” (Armonk, NY, USA) and SAS V9.4 (SAS Institute, Cary, NC, USA).

## Results

The median age at surgery was 65 (IQR, 60–69) years. After a median follow-up of 6.3 [interquartile range (IQR) 4.8–7.6] years, 3% (42) had died. The response rate was 76.8% (936/1218). Responders and non-responders showed no relevant differences in age, oncological risk, Charlson score, or surgical approach. The whole collective was recently published [16]. Therein 404 patients underwent robotic-assisted radical prostatectomy (RARP) und 532 patients open retropubic prostatectomy (ORP). Urinary continence rate was 83% after RARP and 89% after RARP. Potency rate of preoperatively potent patients with nerve-sparing surgery was 47% after RARP and 36% after ORP. In multivariate analysis, there was no difference in functional outcome between both approaches.

### Urinary incontinence

After a median follow-up of 6.3 years after RP, 14% (134/936) of patients were incontinent, and 25% (26/104, 30 missing) of these patients underwent incontinence surgery. Forty-six percent (12/26) of these patients received slings, 12% (3/26) received systems (ATOMS or ProAct), 38% (10/26) received artificial sphincters and 15% (4/26) received other surgical procedures. Three of 26 (12%) patients underwent multiple surgeries. Comparing incontinent patients who did and did not undergo incontinence surgery, there was no difference in age (65.8 ± 8.8 vs. 66.7 ± 6.1, *p* = 0.6), Charlson score (2 + 81% vs. 86%, *p* = 0.2) or d’Amico score (high risk 42 vs. 43%, *p* = 0.9). Patients who underwent surgery used fewer pads (≥ 2 pads 42 vs. 100%, *p* < 0.001) and showed a better EPIC urinary incontinence score (24.2 ± 17.6 vs. 54.4 ± 37.8, *p* < 0.001) (Suppl Table 1).

41% (31/75, 3 missing) of UI patients indicated moderate-to-severe disease. Analysing this subpopulation, there was no difference in age (66.8 ± 5.8 vs. 66.7 ± 6.6, *p* = 1.0), Charlson score (2 + 86% vs. 86%, *p* = 0.7) or d’Amico score (high risk 40 vs. 52%, *p* = 0.3) compared to the whole cohort. Patients moderately to severely affected by UI showed worse mental health (PHQ-4 Depression score (1.3 ± 1.3 vs. 0.6 ± 0.9, *p* = 0.02), PHQ-4 anxiety score (1.4 ± 1.4 vs. 0.6 ± 1.0, *p* = 0.007), PHQ-4 total score (2.8 ± 2.7 vs. 1.2 ± 1.5, *p* = 0.005)) and worse quality of life (EORTC Global Health score (50.3 ± 20.0 vs. 68.2 ± 19.9, *p* < 0.001), EORTC Social Functioning score (48.9 ± 25.1 vs. 85.2 ± 17.7, *p* < 0.001)). These patients also had a worse EPIC urinary incontinence score (13.4 ± 14.0 vs. 31.7 ± 16.4, *p* < 0.001) and EPIC urinary irritative symptoms score (67.0 ± 14.3 vs. 84.9 ± 12.7, *p* < 0.001) (Table 1).

**Table 1:** Incontinent patients without surgery by distress regarding their UI (*n* = 75)

Variable	All (*n* = 75)	No to small problem (*n* = 44)	Moderate to large problem (*n* = 31)	*p* value
Age (years) [mean ± standard deviation, median (IQR)]	66.7 ± 6.1 67.0 (51.0–77.0)	66.8 ± 5.8 67.0 (57.0–77.0)	66.7 ± 6.6 67.0 (51.0–76.0)	1.0
Age adjusted Charlson score (2 missing)				
0	0 (0%)	0 (0%)	0 (0%)	0.7
1	10 (14%)	6 (14%)	4 (14%)	
2 +	63 (86%)	38 (86%)	25 (86%)	
D’Amico score (1 missing)				
Low	13 (17%)	10 (23%)	3 (10%)	0.3
Intermediate	28 (38%)	16 (37%)	12 (39%)	
High	33 (45%)	17 (40%)	16 (51%)	
Internet usage				
Daily	29 (39%)	18 (41%)	11 (35%)	0.8
At least once per week	13 (17%)	7 (16%)	6 (19%)	
Rare	9 (12%)	4 (9%)	5 (16%)	
No internet	24 (32%)	15 (34%)	9 (29%)	
PHQ-4 depression	0.9 ± 1.1 0.5 (0.0–4.0)	0.6 ± 0.9 0.0 (0.0–3.0)	1.3 ± 1.3 1.0 (0.0–4.0)	**0.02**
PHQ-4 anxiety	0.9 ± 1.2 0.0 (0.0–5.0)	0.6 ± 1.0 0.0 (0.0–4.0)	1.4 ± 1.4 1.0 (0.0–5.0)	**0.007**
PHQ-4 total	1.8 ± 2.2 1.0 (0.0–9.0)	1.2 ± 1.5 0.0 (0.0–5.0)	2.8 ± 2.7 2.0 (0.0–9.0)	**0.005**
EORTC global health	60.8 ± 21.7 66.7 (0.0–100.0)	68.2 ± 19.9 66.7 (0.0–100.0)	50.3 ± 20.0 50.0 (16.7–83.3)	**< 0.001**
EORTC social functioning	70.5 ± 27.5 66.7 (0.0–100.0)	85.2 ± 17.7 91.7 (33.3–100.0)	48.9 ± 25.1 50.0 (0.0–100.0)	**< 0.001**
EPIC urinary continencet	24.1 ± 17.8 22.8 (0.0–75.0)	31.7 ± 16.4 29.0 (6.3–75.0)	13.4 ± 14.0 8.3 (0.0–56.3)	**< 0.001**
EPIC urinary irritative symptoms	77.9 ± 15.9 81.3 (37.5–100.0)	84.9 ± 12.7 87.5 (62.5–100.0)	67.0 ± 14.3 68.8 (37.5–100.0)	**< 0.001**

Bold values denote statistical significance at the p < 0.05 level

### Erectile dysfunction

81% (755/936) of patients were impotent after RP, and 7% (63/936) indicated no impotence after RP but were using ED treatment. Of all evaluable patients with ED, 40% (319/793) used ED treatment regularly or tried it at least once. Of these patients, 75% (239/319) used oral PDE5 inhibitors, 10% (32/319) used intraurethral medications, 17% (55/319) used penile injection therapy, 38% (121/319) used vacuum erection devices, and no patient underwent surgery for penile prostheses. There was no difference in usage of antiandrogen (5 vs. 8%; *p* = 0.08).

**Fig. 1:**
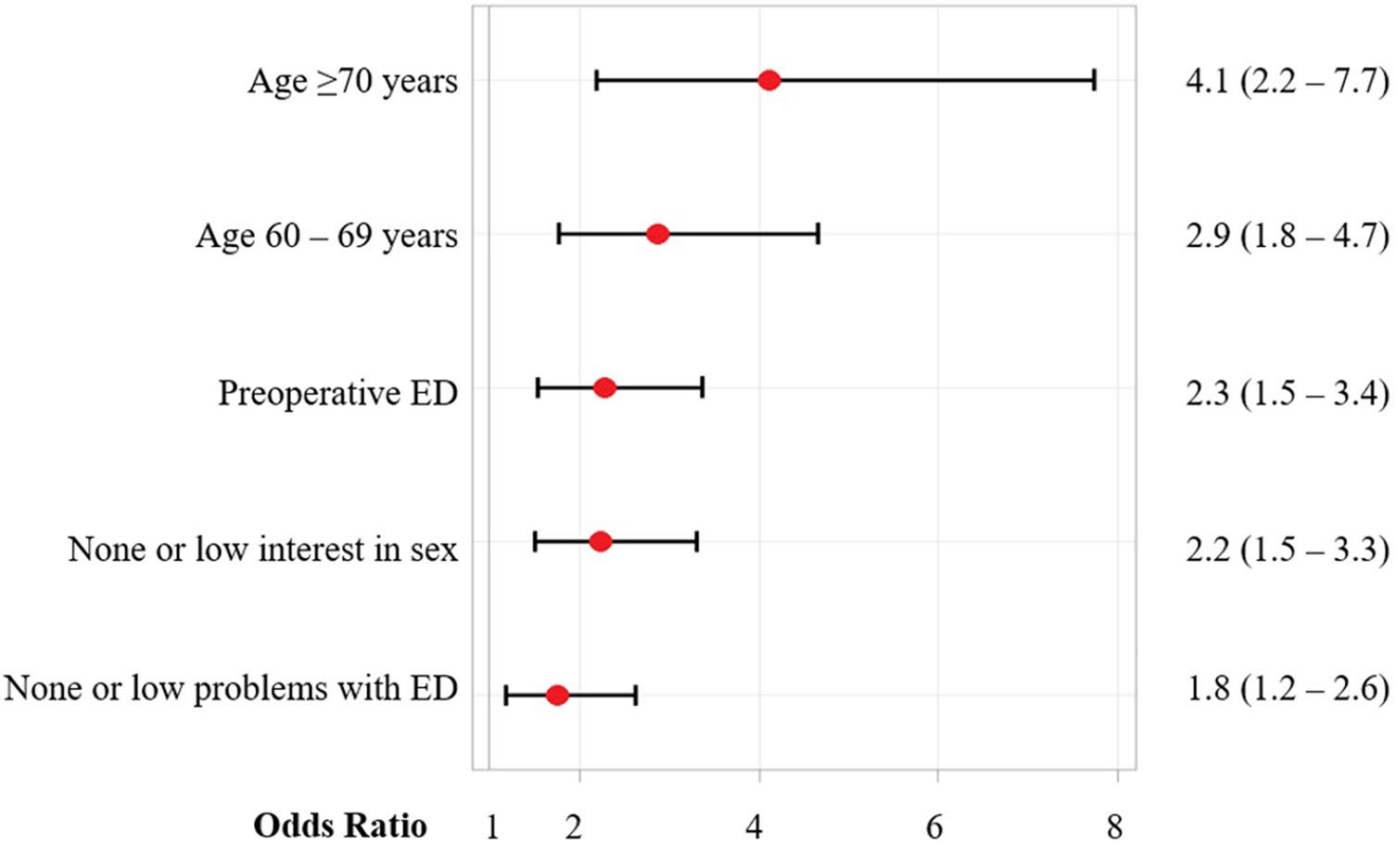
Multivariate analysis of patients not using ED treatment

A total of 64% (499/780) of ED patients were still interested in sex, and 49% (243/499) of them never tried ED treatments. These patients were older (65.8 ± 5.1 vs. 61.8 ± 6.6 years, *p* < 0.001), had a higher Charlson score (2 + : 89 vs 67%, *p* < 0.001), had fewer problems with impaired sexual function (70 vs. 57%, *p* = 0.003) and used the Internet less often for health issues (66 vs. 78%, *p* = 0.03) (Suppl. Table 2). In multivariate analysis, patients not using ED treatments were older [60–69 years OR 2.9 (1.8–4.7), *p* < 0.001; 70 years OR 4.1 (2.2–7.7), *p* < 0.001), had preoperative ED more often (OR 2.3 (1.5–3.4), *p* < 0.001), were less interested in sex (OR 2.2 (1.5–3.3), *p* < 0.001) and reported fewer problems regarding their ED (OR 1.8 (1.2–2.6), *p* = 0.006) (Fig. 1).

Nevertheless, 30% (73/240, 3 missing) of patients with ED not using ED treatments, but with an active interest in sex reported moderate to large problems because of their ED. Patients with moderate to large problems because of their ED showed worse mental health [PHQ-4 depression score (1.1 ± 1.3 vs. 0.4 ± 0.7, *p* < 0.001), PHQ-4 anxiety score (0.9 ± 1.2 vs. 0.4 ± 0.7, *p* = 0.005), PHQ-4 total score (1.9 ± 2.4 vs. 0.8 ± 1.3, *p* < 0.001)] and worse quality of life [EORTC Global Health score (68.7 ± 19.9 vs. 78.5 ± 16.2, *p* < 0.001), EORTC social functioning score (75.3 ± 26.5 vs. 91.6 ± 13.8, *p* < 0.001)]. These patients also had a worse EPIC sexual function score (18.2 ± 18.0 vs. 36.1 ± 19.9, *p* < 0.001) (Table 2).

**Table 2:** Patients with ED, active interest in sex, and without ED treatment by distress regarding their sexual function (*n* = 240)

Variable	All (*n* = 240)	No or small problem (*n* = 167)	Moderate or big problem (*n* = 73)	*p* value
Age (years) [mean ± standard deviation, median (IQR)]	65.8 ± 5.1 66.0 (48.0–77.0)	65.9 ± 5.1 66.0 (51.0–77.0)	65.7 ± 5.2 66.0 (48.0–76.0)	0.7
Age adjusted Charlson score (5 missings)				
0	1 (1%)	0 (0%)	1 (1%)	**0.04**
1	24 (10%)	20 (12%)	4 (6%)	
2 +	210 (89%)	144 (88%)	66 (93%)	
D’Amico score (1 missing)				
Low	69 (28%)	51 (31%)	18 (25%)	0.6
Intermediate	85 (36%)	59 (35%)	26 (36%)	
High	85 (36%)	57 (34%)	28 (39%)	
Preoperative potency				
Potent	74 (31%)	52 (31%)	22 (30%)	0.9
Impotent (or missing)	166 (69%)	115 (69%)	51 (70%)	
Nerve-sparing (10 missing)				
Yes	143 (62%)	102 (64%)	41 (58%)	0.7
No	55 (24%)	36 (23%)	19 (27%)	
Unknown	32 (14%)	21 (13%)	11 (15%)	
Internet usage (1 missing)				
Daily	120 (50%)	87 (52%)	33 (45%)	0.5
At least once per week	38 (16%)	26 (16%)	12 (16%)	
Rare	26 (11%)	19 (11%)	7 (10%)	
No internet	55 (23%)	34 (21%)	21 (29%)	
PHQ depression	0.6 ± 1.0 0.0 (0.0–5.0)	0.4 ± 0.7 0.0 (0.0–3.0)	1.1 ± 1.3 0.0 (0.0–5.0)	**< 0.001**
PHQ anxiety	0.5 ± 0.9 0.0 (0.0–5.0)	0.4 ± 0.7 0.0 (0.0–3.0)	0.9 ± 1.2 0.0 (0.0–5.0)	**0.005**
PHQ total	1.1 ± 1.8 0.0 (0.0–10.0)	0.8 ± 1.3 0.0 (0.0–5.0)	1.9 ± 2.4 1.0 (0.0–10.0)	**< 0.001**
EORTC global health	75.5 ± 18.0 83.3 (16.7–100.0)	78.5 ± 16.2 83.3 (16.7–100.0)	68.7 ± 19.9 66.7 (16.7–100.0)	**< 0.001**
EORTC social functioning	86.6 ± 20.1 100.0 (0.0–100.0)	91.6 ± 13.8 100.0 (50.0–100.0)	75.3 ± 26.5 83.3 (0.0–100.0)	**< 0.001**
EPIC sexual function	30.6 ± 21.0 26.3 (0.0–94.5)	36.1 ± 19.9 34.7 (8.3–94.5)	18.2 ± 18.0 12.5 (0.0–61.2)	**< 0.001**

Bold values denote statistical significance at the p < 0.05 level

## Discussion

This study evaluated the utilisation of care for post-prostatectomy UI and ED in a large cohort of patients undergoing routine care in Germany. 25% (26/104) of incontinent patients underwent surgery to improve continence. Of the remaining patients without surgery, 41% (31/75) reported a moderate to large problem concerning their incontinence with worse mental health and quality of life (*p* < 0.001). A total of 49% (243/499) of ED patients still interested in sex never used ED treatments. 30% (73/240, 3 missing) of these patients not using ED treatment had moderate to large problems because of their ED. They reported worse mental health and quality of life than patients with no or small problems (*p* < 0.001).

There are few studies examining the use of incontinence surgery after radical prostatectomy. Kim et al. examined this topic in a large US population-based study (SEER registry) with 16,348 men after RP [17]. The data correspond with our results concerning the relative frequency of different incontinence procedures. Kim et al. showed that after RP, only 6% of patients underwent incontinence surgery. Because of this low percentage, they assumed an underuse of beneficial procedures. In our study, only 2% (26/936) of all RP patients underwent incontinence surgery. In contrast to Kim et al., we can show very detailed data concerning urinary continence and quality of life. With this profound picture of long-term functional outcomes [16, 18], we can confirm the assumption that there is an underuse of incontinence surgery more securely. However, there are patients with UI using two or more pads per day who are still comfortable with their situation. In our study, 56% (44/78) of incontinent patients without incontinence surgery reported no or only small distress because of their incontinence. However, 31/78 (44%) patients reported moderate-to-great distress because of their incontinence. In the Scandinavian Prostate Cancer Group-4 study, 68% (48/71) of patients with urinary leakage reported moderate-to-great distress because of their incontinence symptoms [19]. Analysing mental health and quality of life in our study shows that 44% (31/78) of patients without surgery who reported moderate-to-great distress had worse scores for depression, anxiety, global health, and social functioning. Therefore, there is an urgent need for improving urinary continence in these patients. Considering the population-based data from the USA [17], this problem seems to exist beyond the German health care system. In our cohort, there was no difference in age (*p* = 0.6) or age-adjusted Charlson score (*p* = 0.2) between incontinent patients with and without incontinence surgery. Therefore, no medical reasons, such as high age or severe comorbidity, explain the lack of surgical treatment. There are several studies indicating insufficient or problematic communication between physicians and patients, including a relevant discrepancy in patient’s perception [20] or significant differences between physician and patient assessments of urinary function [21]. However, these communication issues might be only part of the problem. Inherent health care system factors may also impose restrictions on access and supply of adequate treatment. It is essential to explore these reasons for insufficient utilisation of care to correct them specifically.

Numbers concerning ED treatment use vary widely. A German survey study with 642 patients after RP showed a similar use pattern with regard to the dominance of oral medication (39%) and the low number of penile prostheses (0.3%) [22]. In our study, we showed a much higher usage of oral medication (75%; 239/319), which should be the result of studies of on-demand or regular use of PDE5 inhibitors after nerve-sparing RP [23] and lower prices for oral medication in comparison to 2006, when the mentioned study was published. Surprisingly, there were no patients who underwent penile prosthesis implantation in our nationwide population of 818 patients. An analysis of the US SEER-Medicare database has already shown an underutilisation of penile prostheses, with only 2.3% utilisation after RP [24].

In a retrospective US single-centre study, Miller et al. also discussed a deficiency in ED treatment utilisation [13]. Herein, 31% of patients suffering from ED after RP never received ED treatment. In our study, we identified some plausible parameters associated with not using ED treatment: older age, less interest in sex and fewer problems with ED. A large multicentre study from six countries identified 4622 patients with ED and showed that older age and less interest in sex were barriers to seeking help [25]. In particular, older men lose their desire for treatment and cope with ED as a natural part of ageing. This coping strategy might be a good solution for some of these patients but it is important to identify and motivate men who do suffer from ED. In our study, 30% (73/240) of patients with ED and an active interest in sex reported a significantly worse quality of life and higher scores for depression and anxiety. Therefore, there is an urgent need for treatment in this population. Miller et al. reported that 24% of US patients who complained about moderate to large problems because of their ED and had not received treatment [13]. With a similar percentage, the insufficient utilisation of care for ED patients appears to be an issue across borders. Analogous to the deficit in incontinence surgery utilisation, there may be communication problems between physicians, patients, and their spouses concerning ED [26]. A German study showed that only 53% of post-prostatectomy patients were asked about their erectile function during follow-up care [27]. Often, the Internet is the primary source of information for these patients [28]. Another German survey of 642 patients after RP showed that urologists estimated that 45% of their patients wanted no treatment for ED, while only 29% of the patients actually did not want any kind of ED treatment [22]. These numbers illustrate a missed opportunity for better care, as patients suffering from ED after RP are closely tied to their urologists during aftercare appointments. Nevertheless, further studies are needed to investigate specific reasons for insufficient access to ED treatment.

In conclusion, similar findings of insufficient utilisation of incontinence and ED treatment were also found in the US. But the financial aspect as a reason for this lack of utilisation may differ because of different health care systems. In the US, there exist greater differences in insurance status and available financing of health care because of a lack of a uniform national health care system. In Germany, the health care system is publicly financed, and every insured person is entitled to the same standard of medical care. Incontinence surgery, vacuum erection devices, and penile prosthesis are covered by health insurance. However, medical treatment of erectile dysfunction is not covered by public health insurance creating a small financial barrier for drug treatment. Nowadays prices have become relatively low; for example, four tablets sildenafil cost 12–15 €. Therefore, the economic component should be much more relevant in the US.

There are several limitations to our study. Although we have been able to build on a health care services research project, recruitment is likely to have included some non-responder bias. Patients who participated in this survey are likely to have a higher level of health awareness than non-responders. Moreover, we did not assess the current relationship status and further socioeconomic parameters like the education level. A multivariate analysis within the subset of incontinent patients was not possible because of the small total number. The long follow-up time of 6 years possibly influenced personal attitudes towards impaired functional outcome after RP. On the other hand, this longer interval enabled a validated coverage of patients concerns. This is the first study investigating the utilisation of care for post-prostatectomy incontinence and ED in combination with influences on mental health and quality of life in a large and moderately selected cohort from routine care facilities in Germany. Our study population underwent RP in 114 different institutions representing one-fourth of all German providers of RP. Therefore, the bias of individual practice patterns possibly influencing the treatment of incontinence and ED is very low.

## Conclusion

Half of UI patients never utilising treatment for post-prostatectomy UI reported moderate to large problems because of their impairment with a significant decrease in mental health and quality of life. One-third of impotent patients interested in sex but never trying ED treatment reported moderate to large problems because of their ED with a significant decrease in mental health and quality of life. This indicates insufficient utilisation of care in German cancer survivors. Next, we need to explore the reasons for this insufficient utilisation of care. With this improved understanding, we hopefully will develop interventions to raise the level of care for post-prostatectomy urinary incontinence and ED.

## Electronic supplementary material

Below is the link to the electronic supplementary material.

Supplementary file1 (DOCX 17 KB)

Supplementary file2 (DOCX 18 KB)
