# Supplementary material for: Treatment of post-prostatectomy urinary incontinence and erectile dysfunction: there is insufficient utilisation of care in German cancer survivors
Source: World J Urol. 2020 Dec 1;39(8):2929–36. doi: 10.1007/s00345-020-03526-z (PMC8405514; doi:10.1007/s00345-020-03526-z)
Supplement: Supplementary file 1 — Supplementary file1 (DOCX 17 KB) [file 345_2020_3526_MOESM1_ESM.docx]

**Supplementary Table 1:** Comparison of patients with incontinence who underwent surgery and who underwent no surgery (n=104).

| Variable | | All  (n=104) | Incontinence surgery  (n=26) | No incontinence surgery  (n=78) | p value |
| --- | --- | --- | --- | --- | --- |
| Age (years) [mean ± standard deviation, median (IQR)] | | 66.5 ± 6.8  67.0 (47.0 – 84.0) | 65.8 ± 8.8  67.0 (47.0 – 84.0) | 66.7 ± 6.1  67.0 (51.0 – 77.0) | 0.6 |
| Age adjusted Charlson score  (2 missings) | 0 | 2 (2%) | 2 (8%) | 0 (0%) | 0.2 |
|  | 1 | 14 (14%) | 3 (12%) | 11 (14%) |  |
|  | 2+ | 86 (84%) | 21 (80%) | 65 (86%) |  |
| D’Amico score  (1 missing) | low | 21 (20%) | 6 (23%) | 15 (19%) | 0.9 |
|  | intermediate | 38 (37%) | 9 (35%) | 29 (38%) |  |
|  | high | 44 (43%) | 11 (42%) | 33 (43%) |  |
| Internet usage | daily | 41 (39%) | 12 (46%) | 29 (37%) | 0.8 |
|  | at least once per week | 19 (18%) | 5 (19%) | 14 (18%) |  |
|  | rare | 13 (13%) | 3 (12%) | 10 (13%) |  |
|  | no internet | 31 (30%) | 6 (23%) | 25 (32%) |  |
| PHQ Depression | | 0.8 ± 1.0  1.0 (0.0 – 4.0) | 0.7 ± 0.8  0.5 (0.0 – 2.0) | 0.9 ± 1.1  1.0 (0.0 – 4.0) | 0.4 |
| PHQ Anxiety | | 0.9 ± 1.3  0.0 (0.0 – 6.0) | 0.7 ± 1.4  0.0 (0.0 – 0.6) | 0.9 ± 1.2  0.0 (0.0 – 5.0) | 0.5 |
| PHQ Total | | 1.7 ± 2.1  1.0 (0.0 – 9.0) | 1.4 ± 1.9  0.5 (0.0 – 8.0) | 1.8 ± 2.2  1.0 (0.0 – 9.0) | 0.4 |
| EORTC Global Health | | 63.3 ± 21.6  66.7 (0.0 – 100.0) | 70.0 ± 21.2  83.3 (16.7 – 100.0) | 61.2 ± 21.4  66.7 (0.0 – 100.0) | 0.08 |
| EORTC Social Functioning | | 72.7 ± 27.6  66.7 (0.0 – 100.0) | 78.8 ± 28.5  100.0 (0.0 – 100.0) | 70.6 ± 27.2  66.7 (0.0 – 100.0) | 0.2 |
| EPIC Urinary Continence | | 31.7 ± 27.3  22.8 (0.0 – 100.0) | 54.4 ± 37.8  58.5 (0.0 – 100.0) | 24.2 ± 17.6  22.8 (0.0 – 75.0) | **<0.001** |
| EPIC Urinary Irritative Symptoms | | 79.5 ± 17.0  81.3 (25.0 – 100.0) | 85.4 ± 19.3  87.5 (25.0 – 100.0) | 77.7 ± 15.9  81.3 (37.5 – 100.0) | 0.07 |
