# Supplementary material for: Treatment of post-prostatectomy urinary incontinence and erectile dysfunction: there is insufficient utilisation of care in German cancer survivors
Source: World J Urol. 2020 Dec 1;39(8):2929–36. doi: 10.1007/s00345-020-03526-z (PMC8405514; doi:10.1007/s00345-020-03526-z)
Supplement: Supplementary file 2 — Supplementary file2 (DOCX 18 KB) [file 345_2020_3526_MOESM2_ESM.docx]

**Supplementary Table 2:** Comparison of patients with ED and interest in sex who tried vs. never used ED treatment (n=499).

| Variable | | All  (n=499) | Tried ED treatment at least once (n=256) | Never tried ED treatment (n=243) | p value |
| --- | --- | --- | --- | --- | --- |
| Age (years) [mean ± standard deviation, median (IQR)] | | 63.8 ± 6.2  64.0 (46.0 – 84.0) | 61.8 ± 6.6  62.0 (46.0 – 84.0) | 65.8 ± 5.1  66.0 (48.0 – 77.0) | **<0.001** |
| Age adjusted Charlson score  (12 missings) | 0 | 10 (2%) | 9 (4%) | 1 (1%) | **<0.001** |
|  | 1 | 97 (20%) | 73 (29%) | 24 (10%) |  |
|  | 2+ | 380 (78%) | 168 (67%) | 212 (89%) |  |
| D’Amico score  (2 missings) | low | 165 (33%) | 94 (37%) | 71 (29%) | 0.1 |
|  | intermediate | 176 (36%) | 91 (36%) | 85 (35%) |  |
|  | high | 156 (31%) | 70 (27%) | 86 (36%) |  |
| Preoperative potency | potent | 216 (43%) | 140 (55%) | 76 (31%) | **<0.001** |
|  | impotent  (or missing) | 283 (57%) | 116 (45%) | 167 (69%) |  |
| Nerve-sparing  (27 missing) | yes | 321 (68%) | 177 (74%) | 33 (14%) | **0.02** |
|  | no | 90 (19%) | 35 (15%) | 55 (24%) |  |
|  | unknown | 61 (13%) | 28 (11%) | 144 (62%) |  |
| Internet usage  (4 missings) | daily | 271 (55%) | 150 (59%) | 121 (50%) | **0.03** |
|  | at least once per week | 85 (17%) | 47 (19%) | 38 (16%) |  |
|  | rare | 48 (10%) | 20 (8%) | 28 (11%) |  |
|  | no internet | 91 (18%) | 36 (14%) | 55 (23%) |  |
| PHQ Depression | | 0.7 ± 1.0  0.0 (0.0 – 6.0) | 0.7 ± 1.1  0.0 (0.0 – 6.0) | 0.6 ± 1.0  0.0 (0.0 – 5.0) | 0.2 |
| PHQ Anxiety | | 0.6 ± 1.0  0.0 (0.0 – 5.0) | 0.6 ± 1.0  0.0 (0.0 – 5.0) | 0.6 ± 1.0  0.0 (0.0 – 5.0) | 0.4 |
| PHQ Total | | 1.3 ± 1.8  0.0 (0.0 – 11.0) | 1.4 ± 1.9  1.0 (0.0 – 11.0) | 1.2 ± 1.8  0.0 (0.0 – 10.0) | 0.2 |
| EORTC Global Health | | 74.6 ± 17.7  83.3 (16.7 – 100.0) | 73.8 ± 17.4  83.3 (16.7 – 100.0) | 75.4 ± 18.0  83.3 (16.7 – 100.0) | 0.3 |
| EORTC Social Functioning | | 84.3 ± 20.5  100.0 (0.0 – 100.0) | 82.2 ± 20.7  83.3 (0.0 – 100.0) | 86.5 ± 20.0  100.0 (0.0 – 100.0) | **0.02** |
| EPIC Sexual Function | | 35.2 ± 23.9  31.6 (0.0 – 95.8) | 39.7 ± 25.5  36.2 (0.0 – 95.8) | 30.5 ± 21.1  26.3 (0.0 – 94.5) | **<0.001** |
